# Association of genetic variants in the promoter region of genes encoding p22phox (*CYBA*) and glutamate cysteine ligase catalytic subunit (*GCLC*) and renal disease in patients with type 1 diabetes mellitus

**DOI:** 10.1186/1471-2350-12-129

**Published:** 2011-09-30

**Authors:** Suzana M Vieira, Maria B Monteiro, Tatiana Marques, Ana M Luna, Maria A Fortes, Márcia Nery, Márcia Queiroz, Sérgio A Dib, Márcio F Vendramini, Mirela J Azevedo, Luis H Canani, Maria C Parisi, Elizabeth J Pavin, Daniel Giannella-Neto, Maria L Corrêa-Giannella

**Affiliations:** 1 Laboratório de Endocrinologia Celular e Molecular (LIM-25), Faculdade de Medicina da Universidade de São Paulo. Av. Dr. Arnaldo, 455 #4305, 01246-903, São Paulo-SP, Brazil; 2 Hospital do Servidor Público Estadual (HSPE), Rua Pedro de Toledo, 1800, 04039-901. São Paulo-SP, Brazil; 3 Divisão de Endocrinologia, Hospital das Clínicas, Faculdade de Medicina da Universidade de São Paulo. R. Dr. Eneas de Carvalho Aguiar, 647, 05403-000, São Paulo-SP, Brazil; 4 Centro de Diabetes, Universidade Federal de São Paulo. R. Cel. Lisboa, 826, 04020-041, São Paulo-SP, Brazil; 5 Divisão de Endocrinologia, Hospital de Clínicas de Porto Alegre, Universidade Federal do Rio Grande do Sul, R. Ramiro Barcelos, 2350, 90035-903, Porto Alegre-RS, Brazil; 6 Divisão de Endocrinologia, Departamento de Clínica Médica, Faculdade de Medicina da Universidade Estadual de Campinas (UNICAMP), Rua Tessália Vieira de Camargo, 126, 13084-971. Campinas-SP, Brazil; 7 Laboratório de Gastroenterologia Clínica e Experimental (LIM 07), Faculdade de Medicina da Universidade de São Paulo, Av. Dr. Arnaldo, 455, # 4384, 01246-903, São Paulo-SP, Brazil

## Abstract

**Background:**

Oxidative stress is recognized as a major pathogenic factor of cellular damage caused by hyperglycemia. NOX/NADPH oxidases generate reactive oxygen species and NOX1, NOX2 and NOX4 isoforms are expressed in kidney and require association with subunit p22phox (encoded by the *CYBA *gene). Increased expression of p22phox was described in animal models of diabetic nephropathy. In the opposite direction, glutathione is one of the main endogenous antioxidants whose plasmatic concentrations were reported to be reduced in diabetes patients. The aim of the present investigation was to test whether functional single nucleotide polymorphisms (SNPs) in genes involved in the generation of NADPH-dependent O_2_^•- ^(-675 T → A in *CYBA*, unregistered) and in glutathione metabolism (-129 C → T in *GCLC *[rs17883901] and -65 T → C in *GPX3 *[rs8177412]) confer susceptibility to renal disease in type 1 diabetes patients.

**Methods:**

401 patients were sorted into two groups according to the presence (n = 104) or absence (n = 196) of overt diabetic nephropathy or according to glomerular filtration rate (GFR) estimated by Modification of Diet in Renal Disease (MDRD) equation: ≥ 60 mL (n = 265) or < 60 mL/min/1.73 m^2 ^(n = 136) and were genotyped.

**Results:**

No differences were found in the frequency of genotypes between diabetic and non-diabetic subjects. The frequency of GFR < 60 mL/min was significantly lower in the group of patients carrying *CYBA *genotypes T/A+A/A (18.7%) than in the group carrying the T/T genotype (35.3%) (P = 0.0143) and the frequency of GFR < 60 mL/min was significantly higher in the group of patients carrying *GCLC *genotypes C/T+T/T (47.1%) than in the group carrying the C/C genotype (31.1%) (*p *= 0.0082). Logistic regression analysis identified the presence of at least one A allele of the *CYBA *SNP as an independent protection factor against decreased GFR (OR = 0.38, CI95% 0.14-0.88, *p *= 0.0354) and the presence of at least one T allele of the *GCLC *rs17883901 SNP as an independent risk factor for decreased GFR (OR = 2.40, CI95% 1.27-4.56, *p *= 0.0068).

**Conclusions:**

The functional SNPs *CYBA *-675 T → A and *GCLC *rs17883901, probably associated with cellular redox imbalances, modulate the risk for renal disease in the studied population of type 1 diabetes patients and require validation in additional cohorts.

## Background

Diabetic nephropathy (DN) represents one of the leading causes of end-stage renal disease. Unlike diabetic retinopathy, with up to 80% incidence in diabetes patients with more than 20 years' diagnoses [1], DN affects approximately one third of type 1 diabetes patients during their lifetime, at times irrespective of the glycemic control [2]. These findings taken together with results of familial studies [3], point to the existence of genetic susceptibility to the renal lesions caused by chronic hyperglycemia.

Oxidative stress is currently recognized as a major pathogenic factor of cellular damage caused by hyperglycemia, being considered the final common pathway through which hyperglycemia-related pathways (polyol, advanced glycation end products, protein kinase C and hexosamine) can trigger the chronic complications of diabetes [4]. NOX/NADPH oxidases catalyse NADPH-dependent reduction of O_2 _to O_2_^•-^, generating reactive oxygen species (ROS). NOX1, NOX2 and NOX4 are isoforms expressed in the kidney [5] whose activation requires association with subunit p22phox, encoded by the *CYBA *gene. Increased expression of p22phox was described in animal models of DN [6].

Acting in the opposite direction, glutathione (GSH) is one of the main endogenous antioxidants whose plasmatic concentrations were reported to be reduced in diabetes patients [7]. GSH synthesis requires the glutamate-cysteine ligase enzyme (γ-GCL) in the first and rate-limiting step for its production. This enzyme is a heterodimer composed by a heavy catalytic subunit, encoded by the *GCLC *gene, as well as a light regulatory subunit [8]. GSH regulates ROS concentrations via reactions catalyzed by glutathione peroxidases (GPX), such as the plasmatic GPX (GPX3) [9].

Given the role of oxidative stress in the etiopathogenesis of diabetic complications, we examined the association between the presence of renal disease in type 1 diabetes patients and functional single nucleotide polymorphisms (SNPs) in the promoter region of three genes related to cellular redox balance: (1) -675 T → A in *CYBA *(unregistered), where the T allele is associated with higher phagocytic NADPH oxidase activity [10], (2) -129 C → T in *GCLC *(rs17883901), where the T allele determines a lower promoter activity [8] and -65 T → C in *GPX3 *(rs8177412), where the C allele is present in a haplotype associated with a lower promoter transcriptional activity [9].

## Methods

A total of 401 type 1 diabetes patients and 188 non-diabetic subjects age and gender matched were included between October 2004 and February 2011. The study was carried out in compliance with the Institutional Ethics Committee and the Declaration of Helsinki, with informed consent being given to each participant. Inclusion criterion was diabetes duration ≥ 10 years. DN status was determined by measurements of urinary albumin-to-creatinine ratio (ACR) or urinary albumin excretion rate (UAER). Patients presenting persistent macroalbuminuria (> 300 mg/g of creatinine or > 200 μg/min) or proteinuria (> 500 mg/24 h) were classified as having overt DN (n = 104) and patients without overt DN (n = 196) presented persistent normoalbuminuria. Patients with microalbuminuria were excluded from this analysis. Patients were also stratified according to Modification of Diet in Renal Disease (MDRD)-estimated GFR: ≥ 60 mL (n = 265) or < 60 mL/min/1.73 m^2 ^(n = 136). All patients presenting autoimmune diseases and HIV or HCV infection were excluded from the study, as well as patients with overt DN or GFR < 60 mL/min/1.73 m^2 ^who did not present diabetic retinopathy.

Hypertension was defined as blood pressure > 140/90 mmHg on two occasions or use of antihypertensive drugs. UAER was measured in 24-h urine samples by immunoturbidimetry and ACR was measured in spot urine samples using nephelometry, in at least two samples collected over the preceding 6 months. HbA_1C_, creatinine, triglycerides, HDL and LDL-cholesterol were measured as described [11].

*GCLC *-129 C → T (rs17883901; location 6:53410037) was determined by PCR-RFLP as previously described [8], *GPX3 *-65 T → C (rs8177412; location 5: 150400087) was genotyped by sequencing of PCR products (5' CCTGACTTCCACCTCTCTGC 3' and 5' CGCCCTCCCCGCTGCTCCTC 3') on an ABI3130DNA sequencer (Applied Biosystems). *CYBA *-675 A → T (unregistered; location 16:88718096) [10] was genotyped by PCR using specific primers (sense: 5'-GCGCTGGCTCACCAC-3' and antisense: 5'-ACTGGGAAAGCACAGAATGCA-3') and fluorescent-labelled probes (*VIC*: 5'-CCTCCCGAACCCAGG-3' and *FAM*: 5'-CCTCCCGTACCCAGG-3') (TaqMan, Applied Biosystems). Genotyping success rates were 100% for the *GCLC *and *GPX3 *SNPs and 98% for the *CYBA *SNP. We achieved 100% concordance in the analysis of duplicate samples (15% of total) for all SNPs (the SNP evaluated by RFLP was double genotyped by direct sequencing).

Statistical analyses were performed using JMP 8.0 (JMP SAS Institute Inc). Pearson's χ^2 ^test was used to compare genotype frequencies between non-diabetic and diabetic groups. Mann-Whitney and Fisher's χ^2 ^test were used to compare continuous and categorical variables, respectively. Magnitude of association was estimated using odds ratios (OR) and adjusted OR was estimated by logistic regression for possible confounders (sex, age at diagnosis, diabetes duration, hypertension, triglyceride and cholesterol levels, and HbA_1C_). P values < 0.05 were considered significant. Power calculations were carried out with the CaTS power calculator [12] and the power of the study was > 80% to detect associations of SNPs (dominant model) with a minor allele frequency ≥ 0.11 and a genotype relative risk ≥ 1.6. Benjamini-corrected false-discovery rate was employed to control for multiple hypothesis testing (MHT) [13].

## Results

The distribution of genotypes was consistent with Hardy-Weinberg equilibrium for all SNPs. No differences were found in the age and sex distribution and in the frequency of genotypes between diabetic and non-diabetic subjects (Table 1). The characteristics of the patients according to GFR status are depicted in Table 2. Patients with GFR < 60 mL/min/1.73 m^2 ^are older, present longer diabetes duration, higher frequency of hypertension and of use of lipid lowering drugs, higher plasmatic concentrations of triglycerides and lower plasmatic concentrations of HDL-cholesterol.

**Table 1:** Frequency of *CYBA *- 657 T → A (unregistered), *GCLC *- 129 C → T (rs17883901) and *GPX3 *- 65 T → C (rs8177412) polymorphisms in type 1 diabetes patients and non-diabetic control subjects.

	Non-diabetic control subjects n = 118	Type 1 diabetes patients n = 401
**Sex (male/female)**	44.2%/54.8%	49.5%/50.5%

Age (years)*	36 (29-44.5)	34.7 (27.8-43)

**- 675 T → A *CYBA *genotypes**		

T/T	101 (85.6%)	351 (87.5%)

T/A	17 (14.4%)	47 (11.7%)

A/A	0 (0%)	3 (0.8%)

**- 129 C → T *GCLC *genotypes**		

C/C	97 (82.2%)	333 (83%)

C/T	21 (17.8%)	64 (16%)

T/T	0 (0%)	4 (1%)

**- 65 T → C *GPX3 *genotypes**		

T/T	87 (73.7%)	298 (74.3%)

T/C	28 (23.8%)	98 (24.5%)

C/C	3(2.5%)	5 (1.2%)

*median ± interquartile interval.

**Table 2:** Characteristics of type 1 diabetes patients according to the Glomerular Filtration Rate (GFR) status.

	GFR ≥ 60 mL/min/1.73 m^2 ^(n = 265)	GFR < 60 mL/min/1.73 m^2^ (n = 136)	*p *value
Sex (male/female) (%)	42/58	50/50	0.1410

**Age (years)**	**33 (25.7-42.8)**	**38 (30.8-45.6)**	**0.003**

Age at diabetes diagnosis (years)	11.5 (6-18)	13 (9-20.7)	0.1206

**Duration of diabetes (years)**	**20.6 (16.1-25.1)**	**23 (18-31)**	**0.0086**

**Arterial hypertension (%)**	**36.7**	**64.5**	**< 0.0001**

**Use of lipid-lowering drugs (%)**	**16.4**	**25.3**	**0.0325**

Use of ACE inhibitors (%)	21.7	21	0.8591

HbA_1_C (%)	8.25 (7.4-9.7)	8.4 (7.4-9.8)	0.7027

Cholesterol (mg/dL)	172 (149-199)	179 (151-211)	0.1437

**HDL cholesterol (mg/dL)**	**58 (49-69)**	**52 (43-64.2)**	**0.0134**

LDL cholesterol (mg/dL)	91 (76-113)	103 (78-129)	0.0634

**Triglycerides (mg/dL)**	**71 (53-107)**	**98 (72-145)**	**< 0.0001**

Data are median ± interquartile interval; ACE: angiotensin converting enzyme; GFR: glomerular filtration rate.

No statistically significant differences were found in frequency of the genotypes T/C+C/C of the SNP *GPX3 *rs8177412 between patients with and without overt DN (29.8% and 24%, respectively) and between patients with GFR < and ≥ 60 mL/min/1.73 m^2 ^(28.4% and 24%, respectively) (data not shown).

The characteristics of the patients according to the genotypes of SNPs *CYBA *-675 A → T and *GCLC *rs17883901 are depicted in Table 3. The frequency of overt DN was significantly lower in the group of patients carrying *CYBA *genotypes T/A+A/A (20%) than in the group carrying the T/T genotype (36.2%) (*p *= 0.0398) but this association lost significance if corrected for MHT. The frequency of GFR < 60 mL/min/1.73 m^2 ^was also significantly lower in the group of patients carrying genotypes T/A+A/A (18.7%) than in the group carrying the T/T genotype (35.3%) (*p *= 0.0143). Regarding *GCLC *rs17883901, the frequency of GFR < 60 mL/min/1.73 m^2 ^was significantly higher in the group of patients carrying genotypes C/T+T/T (47.1%) than in the group carrying the C/C genotype (31.1%) (*p *= 0.0082). Both associations remained significant after correction for MHT. *GCLC *C/T+T/T genotypes were also associated with a significantly lower age at diabetes diagnosis (11 years old) in comparison to the C/C genotype (13 years old) (*p *= 0.0073) and with a higher frequency of use of angiotensin converting enzyme inhibitors (31.4% *versus *18.8%, respectively; *p *= 0.0164).

**Table 3:** Characteristics of type 1 diabetes patients according to the genotypes of *CYBA *-675 T → A (unregistered) or *GCL*C rs17883901 SNPs.

	*CYBA* TT (n = 353)	*CYBA* TA+AA (n = 48)	*P *value	*GCLC* CC (n = 331)	*GCLC* CT+TT (n = 70)	*p *value
Sex (male/female) (%)	45/55	43/57	0.4334	53/47	38/62	0.1259

Age (years)	34.6 (27.7-43)	33 (27.8-43)	0.4778	35 (27.9-44)	34.6 (27.1-42)	0.6965

**Age at diabetes diagnosis (years)**	12 (7-19)	13 (10-19)	0.3466	**13 (7.5-20)**	**11 (7-15.2)**	**0.0073**

Duration of diabetes (years)	21 (16.4-26)	20.3 (16-27)	0.7917	21 (16.6-26)	21.6 (17-29.4)	0.6799

Arterial hypertension (%)	45.6	44.9	0.5250	46.1	43.4	0.3937

Use of lipid-lowering drugs (%)	19	15.7	0.3583	17.4	25.3	0.1336

**Use of ACE inhibitors (%)**	21.1	18.3	0.4068	**18.8**	**31.4**	**0.0164**

HbA_1_C (%)	8.4 (7.4-9.6)	8.25 (7.2-10)	0.6329	8.3 (7.3-9.8)	8.3 (7.3-9.3)	0.9669

Cholesterol (mg/dL)	173 (150-200)	180 (149.5-209)	0.4319	174 (149-201)	174 (157-197)	0.7560

HDL cholesterol (mg/dL)	55 (46-67)	58 (47-69)	0.2954	57 (47-68)	52 (43-64.5)	0.0986

LDL cholesterol (mg/dL)	95 (78-118)	97 (75.2-124.4)	0.7686	94.4 (78-119)	94.2 (78-116)	0.8902

Triglycerides (mg/dL)	82 (58-118)	73 (54-113)	0.4712	80 (57-119.2)	85.5 (58-114.2)	0.3683

**Overt DN (%)**	**36.2**	**20**	**0.0398**	33.6	40	0.2387

**GFR < 60 mL/min (%)**	**35.3**	**18.7**	**0.0143**	**31.1**	**47.1**	**0.0082**

Data are median ± interquartile interval; ACE: angiotensin converting enzyme; DN: diabetic nephropathy; GFR: glomerular filtration rate.

Logistic regression analysis with GFR < 60 mL/min/1.73 m^2 ^as dependent variable identified the presence of at least one A allele of the *CYBA *SNP as an independent protection factor against decreased GFR (OR = 0.38, CI95% 0.14-0.88, *p *= 0.0354) and the presence of at least one T allele of the *GCLC *rs17883901 SNP as an independent risk factor for decreased GFR (OR = 2.40, CI95% 1.27-4.56, *p *= 0.0068). Longer diabetes duration, arterial hypertension and higher triglycerides concentrations were also identified as independent risk factors for a GFR < 60 mL/min/1.73 m^2 ^(Table 4).

**Table 4:** Logistic regression analyses with glomerular filtration rate < 60 mL/min/1.73 m^2 ^as dependent variable.

	*OR*	*CI 95*	*p value*
Age at diabetes diagnosis	1.52	0.90-2.59	0.1139

**Duration of diabetes**	**2.08**	**1.22-3.59**	**0.0075**

Female sex	1.35	0.80-2.27	0.2521

**Arterial hypertension**	**1.98**	**1.18-3.33**	**0.0098**

HbA_1_C	1.19	0.70-2.03	0.5060

Cholesterol concentrations	1.51	0.89-2.56	0.1256

**Triglyceride concentrations**	**2.30**	**1.30-4.10**	**0.043**

***CYBA *(TA+AA)**	**0.38**	**0.14-0.88**	**0.0354**

			

Age at diabetes diagnosis	1.50	0.88-2.56	0.1296

**Duration of diabetes**	**1.98**	**1.16-3.39**	**0.0116**

Female sex	1.50	0.89-2.52	0.1215

**Arterial hypertension**	**1.98**	**1.18-3.33**	**0.0090**

HbA_1_C	1.15	0.68-1.95	0.5792

Cholesterol concentrations	1.39	0.82-2.35	0.2176

**Triglyceride concentrations**	**2.47**	**1.40-4.38**	**0.0018**

***GCLC *(CT + TT)**	**2.40**	**1.27-4.56**	**0.0068**

OR- Odds ratio; CI- confidence interval.

## Discussion

In the current study, the presence of at least one A allele in -675 T → A SNP and at least one T allele in rs17883901 located, respectively, at the promoter region of the *CYBA *and *GCLC *genes modulate the risk for decreased GFR in type 1 diabetes patients even after adjusting for other risk factors.

The participation of NOX-derived ROS in DN has been previously demonstrated in experimental models of diabetes [6,14] while the contribution of genes coding proteins belonging to the NADPH system to genetic predisposition for DN has been little explored. The association of one *CYBA *SNP (+242 C → T [rs4673]) with overt DN was previously demonstrated in type 1 diabetes patients [15]. We evaluated a different functional SNP,-675 T → A and the proportion of patients with GFR < 60 mL/min/1.73 m^2 ^was significantly lower in the group of patients carrying genotypes T/A+A/A. Moreno et al. have previously associated the T allele of this SNP with essential hypertension and with a higher phagocytic NADPH-dependent O_2_^•- ^production in Spanish subjects and they hypothesized that the presence of the T allele increases p22phox transcription by interfering with binding of the transcription factor hypoxia-inducible factor 1-α (HIF-1α) [10]. Because high glucose is able to increase expression of HIF-1α in the glomeruli of animal models of diabetes in conditions of normoxia [16], it is plausible that patients harboring the T/A+A/A genotypes do not present an increase in p22phox transcription rate as high as that observed in patients harboring the T/T genotype in response to HIF-α, which could result in lower ROS generation in conditions of hyperglycemia.

The T allele of the *GCLC *rs17883901 SNP was previously associated to myocardial infarction in the Japanese [8] and Italian [17] populations and to non-alcoholic steatohepatitis in a Brazilian series [18]. No previous studies have associated this SNP with any of the chronic microangiopathic complications of DM, but a study performed in a Swedish population of type 1 diabetes patients found that the C/T genotype was associated with higher anti-GAD autoantibody concentrations compared to the C/C genotype. The authors suggested that this SNP influences anti-GAD levels and perhaps diabetes age at onset [19]. The results of the present study also suggest an effect of this SNP on the age at diabetes onset since age at diabetes diagnosis among those harboring the C/T+T/T genotypes was significantly lower than among patients with the C/C genotype.

Mechanisms underlying the increased susceptibility of type 1 diabetes patients carrying the T allele of the *GCLC *rs17883901 SNP toward the presence of decreased GFR may be the inability to increase concentrations of the enzyme γ-CGS during oxidative stress, which would result in lower production of GSH and thus, increased susceptibility of cells exposed to chronic hyperglycemia to ROS-induced lesions.

This study widens the spectrum of candidate genes for DN belonging to oxidant and antioxidant pathways and corroborates the assumption that therapeutic approaches to improve antioxidant defenses may be a strategy to prevent or delay chronic diabetic complications. However the present findings should be interpreted in the context of limitations of cross-sectional studies and must be validated in larger populations sorted by different phenotypes of renal impairment to further evaluated if these genetic variants are more involved with the decline of GFR than with development of proteinuria or if the current findings only reflect the larger number of patients stratified by GFR than by albuminuria status, given that in this latter analysis, patients presenting normoalbuminuria had been excluded. Besides, the ethnic admixture that characterizes the Brazilian population may be a source of false-positive associations. Ninety percent of the patients of the present study defined themselves as Caucasoid, 7.3% as African descendants and 2.7% as Asian descendants, but previous genetic studies demonstrated that color, as determined by physical evaluation, is a poor predictor of genomic African ancestry in Brazilians [20]. Nevertheless type 1 DM is predominantly a disease of Caucasians and of populations with a great Caucasian genetic admixture [21] and the genotypic frequencies of the *GCLC *and *CYBA *SNPs observed in this Brazilian sample are similar to those reported in the representative Caucasian samples genotyped in the HapMap CEU and in the previous study of the *CYBA *SNP in a Spanish series [10], respectively.

## Conclusions

The functional SNPs -675 T → A in *CYBA *and rs17883901 in *GCLC*, probably associated with cellular redox imbalances, modulate the risk for renal disease in the studied population of type 1 diabetes patients. Furthermore, replication studies for these functional variants will need to be carried out.

## Competing interests

The authors declare that they have no competing interests.

## Authors' contributions

SMV was responsible for samples collection and for organizing the clinical database; MBM and TM were responsible for organizing the DNA bank and performing *CYBA *genotyping; AMCL performed *GCLC *genotyping; MAZF performed *GPX3 *genotyping; MN participated in the design of the study; MQ was co-responsible for organizing the clinical database; SAD provided diabetic patients; MFV provided control subjects and diabetic patients; MJA provided diabetic patients; LHC provided diabetic patients and revised the manuscript; MCP and EJP provided diabetic patients, DGN provided input into the design and analysis for the study and performed the statistical analysis; MLCG participated in the design of the study, obtained funding and wrote the paper. All authors read and approved the final manuscript.

## Pre-publication history

The pre-publication history for this paper can be accessed here:

http://www.biomedcentral.com/1471-2350/12/129/prepub
